# Interim 2025/26 influenza vaccine effectiveness estimates with immuno-epidemiological considerations for A(H3N2) subclade K protection, Canada, January 2026

**DOI:** 10.2807/1560-7917.ES.2026.31.5.2600068

**Published:** 2026-02-05

**Authors:** Lea Separovic, Suzana Sabaiduc, Yuping Zhan, Samantha E Kaweski, Romy Olsha, Maan Hasso, Richard G Mather, Sara Carazo, Christine Lacroix, Isabelle Meunier, Lila N Salhi, James A Dickinson, Nathan Zelyas, Agatha N Jassem, Katie Dover, Charlene Ranadheera, Ruimin Gao, Nathalie Bastien, Danuta M Skowronski

**Affiliations:** 1 British Columbia Centre for Disease Control, Vancouver, Canada; 2 Public Health Ontario, Toronto, Canada; 3 Queen’s University, Kingston, Canada; 4 Institut National de Santé Publique du Québec, Québec, Canada; 5 University of Calgary, Calgary, Canada; 6 Public Health Laboratory, Alberta Precision Laboratories, Edmonton, Canada; 7 National Microbiology Laboratory, Public Health Agency of Canada, Winnipeg, Canada; 8 University of British Columbia, Vancouver, Canada

**Keywords:** Influenza, vaccine effectiveness, test-negative design, A(H3N2), A(H1N1)pdm09, antigenic characterization, imprinting, epidemiology

## Abstract

In interim 2025/26 analyses, the Canadian Sentinel Practitioner Surveillance Network estimates influenza vaccine reduced the risk of medically-attended acute respiratory illness due to predominant influenza A(H3N2) viruses, including antigenically distinct subclade K, by about 40% relative to unvaccinated individuals. Vaccine effectiveness was about 30% against A(H1N1)pdm09, with insufficient case numbers for interim influenza B estimation. Meaningful protection against subclade K, despite substantial vaccine mismatch, is interpreted in the context of immuno-epidemiological considerations, including potential viral glycosylation, imprinting, and pre-immunity effects.

In a pre-season influenza risk assessment publicly posted in October 2025, Canadian investigators used global genomic and antigenic surveillance data to highlight important mutations in circulating influenza A(H3N2) viruses, including glycosylation changes affecting antigenic site A and major substitutions affecting antigenic site B [1]. This viral evolution culminated in sweeping predominance of subclade K, an antigenically distinct variant showing early surge in Europe and elsewhere, and leading to further threat assessments and vaccine mismatch concerns expressed by others [2-4]. Here, the Canadian Sentinel Practitioner Surveillance Network (SPSN) provides interim estimates of 2025/26 influenza vaccine effectiveness (VE), including genotypic and phenotypic characterisation of contributing case viruses and immuno-epidemiological interpretation.

## Epidemiological context

Community-based sentinel practitioners in Canada’s four largest provinces (Alberta, British Columbia, Ontario, Quebec) collected specimens from patients presenting to them with acute respiratory illness (ARI; defined as illness including new or worsening cough potentially due to respiratory infection) within 7 days of onset. Accredited provincial laboratories tested specimens for influenza by commercial or laboratory-developed real-time reverse-transcription PCR. Specimens collected between 26 October 2025 and 10 January 2026 (epidemiological-weeks (epi-weeks): 44–01) from patients ≥ 1 year-old were included in analyses. We based influenza vaccination status on self-report (participant or guardian) [5]. We estimated VE against medically-attended ARI due to laboratory-confirmed influenza using a test-negative design, comparing the odds of testing influenza positive (cases) vs negative (controls) among vaccinated vs unvaccinated participants through the odds ratio (OR), adjusted for confounders. The VE was derived as (1 − OR) × 100%.

Only trivalent influenza vaccine formulations were available in Canada with virtually all publicly-funded vaccines in SPSN provinces being inactivated (≥ 99%) and egg-based (≥ 90% overall). Adjuvanted vaccine was publicly funded for community-dwelling adults aged ≥ 65 years, and in Ontario high-dose vaccines were also provided for them. The A(H3N2) vaccine strain was updated from clade 2a.3a.1 subclade J in 2024/25 to subclade J.2 for 2025/26 [6]. The vaccine strain remained unchanged since 2023/24 for A(H1N1)pdm09 (clade 5a.2a.1, subclades C.1.1 (cell-based) and D (egg-based)), and since 2022/23 for influenza B (Victoria) (clade V1A.3a.2, subclade C) [6].

## Virological characterisation

We sought to whole genome sequence all eligible influenza A case viruses according to provincial or national laboratory protocols as further described in Supplementary Table S1. Haemagglutinin clades and subclades were assigned as per Nextclade [7]. As detailed in Supplementary Table S2, we specify amino acid substitutions in relation to cell-based reference strains, annotating affected antigenic sites and involvement of the receptor binding site (RBS) in parentheses [8]. N-linked glycosylation is defined by the amino acid sequon N-X-T/S, where X may be any amino acid except proline (P), with gain or loss of glycosylation shown as (+/− CHO). We anchor A(H3N2) genetic analyses in relation to the parental subclade J reference strain but interpret mutations in relation to the 2025/26 J.2 vaccine that bears N122D (A)(− CHO), S145N (A) and K276E (C) antigenic site substitutions relative to subclade J, with additional D186A (B) mutation in the egg-adapted high growth reassortant. Canada’s National Microbiology Laboratory antigenically characterised a subset of contributing case viruses by haemagglutination inhibition (HI) assay in relation to the cell-based A(H3N2) and A(H1N1)pdm09 vaccine reference strains [9], defining antigenic distinction (vaccine mismatch) as ≥ 8-fold HI titre reduction [10].

## Virological findings

**Figure 1:**
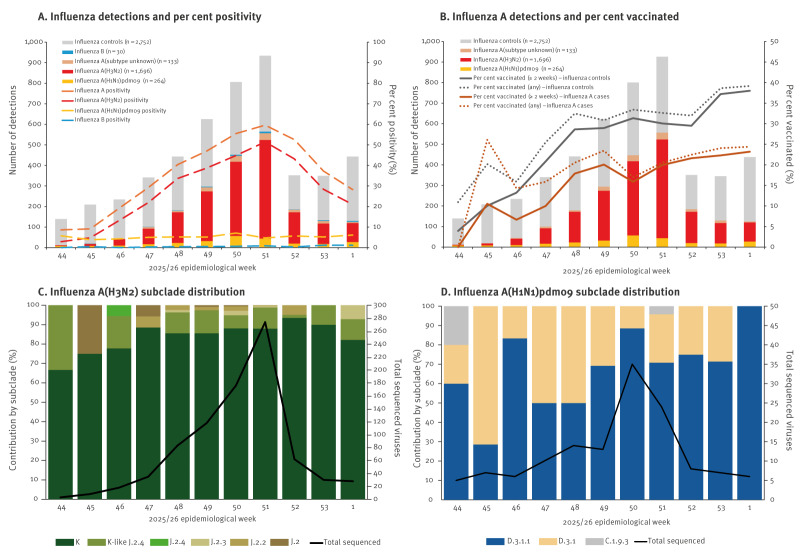
Influenza test-positive cases and test-negative controls, including genetic characterisation, by week of specimen collection, Canadian Sentinel Practitioner Surveillance Network, 26 October 2025–10 January 2026 (epi-weeks 44–01) (n = 4,875)^a^

Overall, 2,121 (44%) of 4,873 included specimens tested influenza positive, almost exclusively influenza A (2,091/2,121; 99%), with one additional influenza A plus B co-infection (Figure 1). Similar to other Canadian national surveillance indicators [11], influenza A per cent positivity peaked in epi-week 51, with steady decline thereafter. Of 1,959/2,092 (94%) subtyped influenza A specimens, 1,694 (86%) were A(H3N2) only, 263 (13%) were A(H1N1)pdm09 only, and two were co-infections (one A(H3N2) plus B; one A(H3N2) plus A(H1N1)pdm09). The subclade distribution of sequenced influenza A case viruses is shown by epi-week in Figure 1 with mutations and provincial distributions detailed in Supplementary Table S2.

Epi-week: epidemiological week.

^a^ Displayed detections in panels A and B include one influenza A(H3N2) and B co-infection and one influenza A(H1N1)pdm09 and A(H3N2) co-infection. These four viruses from two specimens are displayed separately, thus panel A reflects a total of 4,875 influenza test-positive cases and test-negative controls (n = 4,873 when co-infections not counted separately). Both co-infected individuals were unvaccinated.

Influenza test-positive and test-negative specimen tallies by epi-week are shown in panels A and B. Panel A includes all influenza infections (n = 2,123), including two co-infections (one A(H3N2) plus B, one A(H3N2) plus A(H1N1)pdm09), with overlaid per cent positivity (dashed lines). Panel B focuses on influenza A infections (n = 2,093), including two co-infections (one A(H3N2) plus B, one A(H3N2) plus A(H1N1)pdm09),with overlaid per cent vaccinated ≥ 2 weeks before onset (solid line) and per cent vaccinated at any time before onset (dotted line). Subclade distributions and total number of sequenced case viruses (black line) contributing to vaccine effectiveness analyses are shown by epi-week in panel C for influenza A(H3N2) and panel D for influenza A(H1N1)pdm09.

As evident in panel B, a greater fraction of vaccinated cases (15/30) and controls (55/154; 36%) were excluded because still < 2 weeks since vaccination early in the analysis period spanning weeks 44–47, with a lower proportion excluded on that basis during weeks 48–01, comparable among cases (36/407; 9%) and controls (66/706; 9%).

All A(H3N2) subclades displayed in panel C (n = 835 specimens) belong to clade 2a.3a.1. Subclade ‘K-like’ J.2.4 viruses shown in panel C belong to subclade J.2.4 but have acquired most of the additional ‘K-like’ mutations, specifically, S144N (A)(− CHO), N158D (B), and I160K (B). Among displayed A(H1N1)pdm09 subclades in panel D (n = 135), all belong to clade 5a.2a.1 except C.1.9.3 (n = 2), belonging to clade 5a.2a. For further details on virological characterisation, subclade designations, and tallies by province, see Supplementary Table S2.

Of 835/1,696 (49%) sequenced A(H3N2) case viruses, all were clade 2a.3a.1, with most (729/835; 87%) being subclade K, a J.2 descendant variant formerly known as J.2.4.1 and notably differing from the 2025/26 J.2 vaccine by the antigenic site substitutions: T135K (A)(RBS)(– CHO), S144N (A)(+ CHO), N158D (B), I160K (B), Q173R (D), and K189R (B), as well as N145S (A) and A186D (B) owing to vaccine-specific substitutions mentioned above [1,7]. The remaining A(H3N2) viruses (106/835; 13%) were subclade J.2 or its descendants of which nearly three quarters (77/106; 73%) were subclade J.2.4, all but one (76/77; 99%) subclade K-like in sharing the same antigenic site substitutions except Q173R (D).

Antigenic characterisation showed most A(H3N2) viruses were vaccine mismatched (102/112; 91%). Among 91/112 (81%) antigenically characterised viruses that were also genetically sequenced, most (10/12) non-subclade K variants were vaccine matched, except for J.2.3 viruses (2/12) which harbour the additional N158K (B) and K189R (B) mutations relative to the J.2 vaccine strain. All (79/79) subclade K and K-like viruses were vaccine mismatched, including 61/79 (77%) with ≥ 16-fold HI titre reduction (Table 1). 

**Table 1:** Antigenic characterisation of a subset of influenza A(H3N2) viruses included in vaccine effectiveness analysis, Canadian Practitioner Surveillance Network, 26 October 2025–10 January 2026 (epi-weeks 44–01) (n = 112)

Antigenic characteristics	Genetic subclade of test viruses							
	J.2 (n = 4)	J.2.2 (n = 5)	J.2.3 (n = 2)	J.2.4 (n = 1)	K-like J.2.4^a^ (n = 6)	K (n = 73)	No sequence data (n = 21)	Total (n = 112)
**Antigenically similar or distinct (number)**								
Antigenically similar	4	5	0	1	0	0	0	10
Antigenically distinct	0	0	2	0	6	73	21	102
**Fold-differences among antigenically distinct samples (number)**								
8-fold	0	0	0	0	0	18	3	21
16-fold	0	0	1	0	3	49	14	67
≥ 32-fold	0	0	1	0	3	6	4	14

Epi-week: epidemiological week; HI: haemagglutination inhibition.

^a^ Subclade ‘K-like’ J.2.4 viruses belong to subclade J.2.4 but have acquired most of the additional ‘K-like’ mutations, specifically, S144N (A)(− CHO), N158D (B), and I160K (B). For further details on virological characterisation, see Supplementary Table S2.

Antigenic distinction (or vaccine mismatch) was interpreted per convention based upon ≥ 8-fold reduction in HI titre against the case virus when compared with the homologous 2025/26 cell-based J.2 vaccine reference strain against which ferret antisera were originally raised [8].

Of 135/264 (51%) sequenced A(H1N1)pdm09 viruses, almost all (133/135; 99%) were vaccine clade 5a.2a.1, but belonged to descendant subclades D.3.1 (37/133; 28%) and D.3.1.1 (96/133; 72%). Antigenic characterisation showed all but one A(H1N1)pdm09 viruses were vaccine matched (52/53; 98%).

## Epidemiological findings

Participant profiles are displayed in Table 2 for influenza A(H3N2) analyses and in Supplementary Tables S3-S4 for influenza A(H1N1)pdm09 and influenza A overall. The proportion of SPSN controls ≥ 18 years who received the 2025/26 vaccine (744/2,235; 33% without regard to timing) aligns with the last available 2024/25 national vaccine coverage estimates for Canadian adults ≥ 18 years (33%) [12]. Influenza A(H3N2) cases were younger than influenza A(H1N1)pdm09 cases and controls, with median ages of 23 vs 41 and 41 years, respectively (both p < 0.001).

**Table 2:** Participant profile, influenza A(H3N2) analyses, Canadian Sentinel Practitioner Surveillance Network, 26 October 2025–10 January 2026 (epi-weeks 44–01) (n = 4,448)

Characteristics	All ARI participants (column %, unless otherwise specified)						Influenza vaccinated^a^ (row %)					
	Overall		Influenza A(H3N2) cases		Influenza controls		Overall		Influenza A(H3N2) cases		Influenza controls	
	Number	%	Number	%	Number	%	Number	%	Number	%	Number	%
Number (row %)	4,448	100	1,696	38	2,752	62	1,042	23	303	18	739	27
**Age group (years)^b^**												
1–8	784	18	398	23	386	14	125	16	54	14	71	18
9–17	602	14	369	22	233	8	63	10	37	10	26	11
18–49	1,604	36	560	33	1,044	38	271	17	77	14	194	19
50–64	665	15	164	10	501	18	166	25	28	17	138	28
≥ 65	793	18	205	12	588	21	417	53	107	52	310	53
Median (IQR)	35 (13–58)		23 (9–45)		41 (20–62)		57 (31–72)		42 (14–71)		60 (36–72)	
**Sex**												
Female	2,625	59	919	54	1,706	62	651	25	167	18	484	28
Male	1,804	41	772	46	1,032	38	390	22	135	17	255	25
Unknown	19	0	5	0	14	1	1	5	1	20	0	0
**Comorbidity^c^**												
No	3,285	74	1,346	79	1,939	70	581	18	192	14	389	20
Yes	935	21	271	16	664	24	376	40	87	32	289	44
Unknown	228	5	79	5	149	5	85	37	24	30	61	41
**Province**												
Alberta	557	13	238	14	319	12	142	25	42	18	100	31
British Columbia	745	17	166	10	579	21	245	33	36	22	209	36
Ontario	2,297	52	1,032	61	1,265	46	559	24	204	20	355	28
Quebec	849	19	260	15	589	21	96	11	21	8	75	13
**Weeks of specimen collection, 2025/26^d^**												
44–45	330	7	14	1	316	11	24	7	0	0	24	8
46–47	535	12	106	6	429	16	84	16	9	8	75	17
48–49	981	22	391	23	590	21	243	25	73	19	170	29
50–51	1,564	35	841	50	723	26	370	24	148	18	222	31
52–01^e^	1,038	23	344	20	694	25	321	31	73	21	248	36

ARI: acute respiratory illness; IQR: interquartile range; NA: not applicable; SPSN: Sentinel Practitioner Surveillance Network.

^a^ Vaccination status based on participant or guardian report. Participants vaccinated < 2 weeks before onset of symptoms or with unknown vaccination status or timing were excluded. Only trivalent influenza vaccine formulations were used in Canada with virtually all publicly-funded vaccines in SPSN provinces being inactivated (≥ 99% overall; < 5% live-attenuated in British Columbia and Quebec) and egg-based (≥ 90% overall; < 30% cell-based in Alberta and < 5% in Ontario). In all provinces, adjuvanted vaccines were publicly funded for community-dwelling older adults (≥ 65 years, ≥ 75 years in Quebec), with high dose vaccines also publicly funded for them in Ontario.

^b^ Children aged < 1 year excluded based on variability and/or uncertainty in their age-related vaccine eligibility over the course of the season. Older age strata defined as per usual SPSN analyses predicated upon higher likelihood of chronic comorbidity at ≥ 50 years and higher age-associated risk among adults ≥ 65 years [35].

^c^ Includes chronic comorbidities that place individuals at higher risk of serious complications from influenza as defined by Canada’s National Advisory Committee on Immunization [35].

^d^ Missing specimen collection dates were imputed as the date the specimen was received and processed at the laboratory minus 2 days.

^e^ Includes epi-weeks 52, 53, and 01.

**Figure 2:**
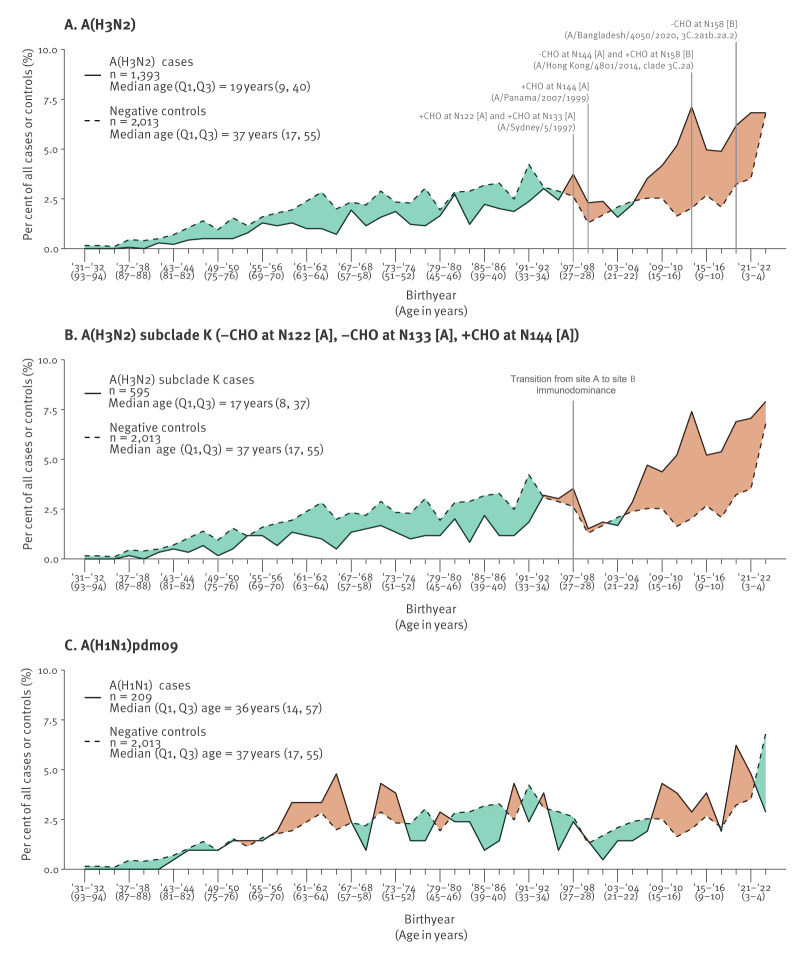
Age and birthyear distributions of unvaccinated influenza cases and controls, by subtype and subclade, Canadian Sentinel Practitioner Surveillance Network, 26 October 2025–10 January 2026 (epi-weeks 44–01) (n = 3,615)

We further explored the age and birthyear distributions of unvaccinated cases, using unvaccinated controls to standardise for age-related sampling differences (Figure 2). This showed a sustained change in the relative proportion of A(H3N2) cases vs controls with inflection point around birthyear 1997, potentially signalling pre-immunity differences. Such striking demarcation in relative case vs control distribution by age was not evident for A(H1N1)pdm09. Overall, adults ≥ 30 years-old (born 1995 and earlier) were significantly under-represented among A(H3N2) cases (517/1,393; 37%), including subclade K (196/595; 33%), relative to controls (1,242/2,013; 62%) (both p < 0.001); whereas, A(H1N1)pdm09 cases aged ≥ 30 years (124/209; 59%) comprised a comparable proportion to controls (p = 0.503).

+ CHO: gain of glycosylation; - CHO: loss of glycosylation; Q1: first quartile; Q3: third quartile

Shown are age and calculated birthyear distributions of unvaccinated cases and unvaccinated controls by 2-year categories; displayed cases include influenza A(H3N2) in panel A, influenza A(H3N2) subclade K only in panel B, and influenza A(H1N1)pdm09 in panel C.

Birthyear is displayed as last two digits (e.g. ’31 for 1931). Birthyear is calculated as 2025 minus age at specimen collection.

Green shading indicates greater contribution by controls than cases; orange indicates the inverse. Overall patterns are unchanged with restriction to controls pan-negative on multiplex respiratory pathogen assays (data not shown).

Sustained gains (+ CHO) and losses (– CHO) of glycosylation over the past 30 years are annotated in panel A, with affected antigenic sites shown in square brackets and representative strains shown in rounded brackets. Other glycans acquired or lost from 1968 to 1995 are not displayed because not expected to impact the transition from antigenic site A to site B immunodominance (panel B). Gains and losses of glycosylation acquired in subclade K, relative to parental strain subclade J, are denoted in the panel B figure title. Of note, the J.2 vaccine shares the – CHO at N122.

**Figure 3:**
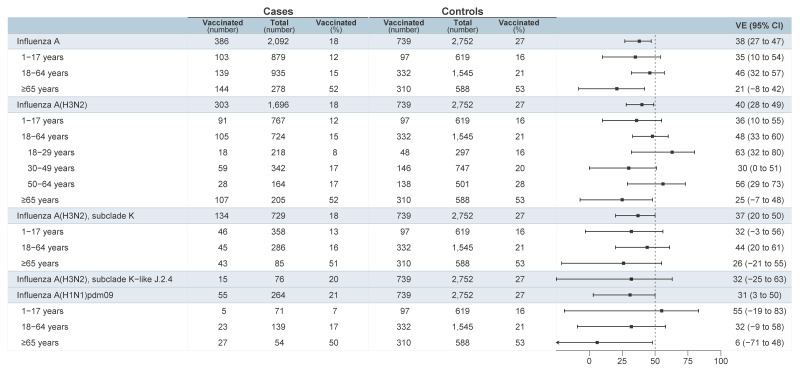
Vaccine effectiveness against influenza A, stratified by age, subtype, and subclade, Canadian Sentinel Practitioner Surveillance Network, 26 October 2025–10 January 2026 (epi-weeks 44–01) (n = 4,844)

CI: confidence interval; epi-week: epidemiological week; OR: odds ratio; VE: vaccine effectiveness.

The adjusted VE estimates against medically-attended outpatient ARI due to influenza A are shown in Figure 3. The VE against A(H3N2) was 40% (95% confidence interval (CI): 28 to 49) overall, driven by predominant subclade K contribution. Restricted to sequenced viruses, VE was similar for subclade K at 37% (95% CI: 20 to 50) and K-like J.2.4 viruses at 32% (95% CI: – 25 to 63). Influenza A(H3N2) VE estimates are stratified according to standard age categories for ease of comparison with estimates elsewhere, including 1–17 years (36%; 95% CI: 10 to 55) and 18–64 years (48%; 95% CI: 33 to 60), each of which were also similar with restriction to subclade K viruses (Figure 3). We explored further age stratification of A(H3N2) estimates hinged at 30 years, suggesting higher VE among adults 18–29 years old (63%; 95% CI: 32 to 80) than 30–49 years old (30%; 95% CI: 0 to 51), but comparable among adults 50–64 years old (56%; 95% CI: 29 to 73). The VE was lowest among adults aged ≥ 65 years (25%; 95% CI: – 7 to 48) but with overlapping CIs throughout. Finally, we estimated VE of 31% (95% CI: 3 to 50) against A(H1N1)pdm09, noting the particularly low sample size and wide CIs with age stratification.

VE was calculated as (1 − OR) × 100%. ORs compare per cent positivity between vaccinated and unvaccinated participants using logistic regression. Firth’s logistic regression was explored as needed to address small sample size; VE estimates differed by ≤ 4% (data not shown).

Adjusted for age group (1–8, 9–17, 18–49, 50–64, ≥ 65 years), calendar time (epi-week), and province (Alberta, BC, Ontario, Quebec). Additional adjustment for sex and/or comorbidity had <10% absolute impact on VE findings, greater but with added uncertainty given reduced sample size upon some stratification (e.g. further reducing point estimates among ≥ 65-year-olds by 18–23% for A(H1N1)pdm09) (data not shown).

Vaccination status per participant/guardian report. Participants vaccinated < 2 weeks before symptom onset or with unknown vaccination status were excluded.

Subclade ‘K-like’ J.2.4 viruses belong to subclade J.2.4 but have acquired most of the additional ‘K-like’ mutations, specifically, S144N (A)(− CHO), N158D (B), and I160K (B). For further details on virological characterisation, see Supplementary Table S2.

Displayed cases include two co-infections (one influenza A(H3N2) plus B, one influenza A(H3N2) plus A(H1N1)pdm09). The influenza A(H3N2) and A(H1N1)pdm09 co-infection was counted in both influenza A(H1N1)pdm09 and A(H3N2) analyses. The co-infected individuals were unvaccinated.

## Discussion

Between November and January of the 2025/26 season, the Canadian SPSN estimates that the risk of medically-attended influenza A(H3N2) illness was reduced by 40% among vaccinated relative to unvaccinated individuals. With about one in two of all contributing case viruses sequenced, we estimate similar subclade K specific VE of 37%. In the context of a J.2 vaccine strain mismatched to the predominantly circulating subclade K variant, we propose agent−host interactions to explain the observed vaccine protection and age-related variation, including potential viral glycosylation, imprinting, and pre-immunity effects.

Since 2012/13, the SPSN has published eight mid-season estimates of influenza A(H3N2) VE spanning epi-week 44 through as late as epi-week 5: two were < 20%, three ranged 40–45%, and three ranged 54–62%, placing the current season’s estimate in the low mid-range [13]. As shown in Supplementary Table S5, our 2025/26 interim A(H3N2) estimates are lower than early reports from England (weeks 40–44 [14]) and Europe (weeks 41–49 [15]). Nonetheless, all VE estimates to date may be higher than anticipated from virological assessments. Conventional antigenic characterisation of SPSN case viruses shows, as elsewhere [14,16], substantial antigenic distinction between subclade K and the 2025/26 cell-based vaccine strain. Additional data suggest even greater antigenic distinction from the egg-based J.2 vaccine strain likely owing to the additional D186A mutation acquired through egg-adaptation of the high-growth reassortant used in inactivated vaccines [14,16]. We note that the cold adapted strain used by the 2025/26 live attenuated influenza vaccine (LAIV) belongs instead to subclade J.2.2 with the clade defining substitution S124N (A) distinguishing it from both the J.2 vaccine and subclade K. Although also egg-based, LAIV does not possess the D186A mutation, having instead acquired two other egg-adaptation mutations with uncertain impact (S219Y (D), I226M (D, RBS)) [17]. The extent to which the higher 2025/26 A(H3N2) VE reported from England, particularly in children (Supplementary Table S5), may reflect greater LAIV use warrants further consideration [14], as does the use of non-egg-based vaccines lacking the D186A mutation more generally.

First-infection ferret antisera used in conventional antigenic characterisation assessments do not capture the complexity of accumulated human influenza virus exposures and immunological histories. In preliminary analysis of 277 human sera collected from Hong Kong hospital patients in November 2025 (unspecified ages or vaccine histories), the proportion with detectable neutralising titres against J.2.2 (52%) were reported to exceed subclade K (18%), lower for threshold titres ≥ 40 (27% and < 1%, respectively) [18]. In a January 2026 preprint, Liu et al. reported egg-based J.2 vaccine immunogenicity against subclade K among 76 individuals aged 24–81 years vaccinated in October or November 2025 [19]. The proportion with HI titres ≥ 40 increased from 11% (8/76) pre- to 39% (30/76) post-vaccination, aligning with VE estimates reported here and suggesting substantial cross-reactive responses against subclade K; however, geometric mean titres were low both pre- (16) and post-vaccination (29), complicating interpretation. While genetic, antigenic and/or serologic studies may be helpful intermediaries to track viral evolution and anticipate vaccine performance and other impacts, epidemiological data are needed for confirmation and complete understanding.

The first influenza infection of childhood induces a robust and long-lasting immunological imprint that is reinforced across the lifespan through re-exposure and boosting of memory responses to epitopes shared with subsequent influenza infections [20]. Changes in viral glycosylation can modulate imprinted responses through selective shielding or exposing of pivotal epitopes, potentially altering hierarchical antigenic site immunodominance [21]. Since 1968, the globular head of A(H3N2) viruses has accumulated N-linked glycans at ca 5–7-year intervals, reaching a functional limit of seven glycans by 2004 [22]. During the late 1990s, following prolonged site A immunodominance, three glycans were added in rapid succession to antigenic site A beginning with A/Sydney/5/1997 acquiring glycans at N122 and N133, followed by A/Panama/2007/1999 acquiring glycosylation at N144 (Figure 2). This heightened glycan shielding of site A shifted antibody immunodominance among subsequent birth cohorts towards antigenic site B [23-25]. In 2014/15, A/Hong Kong/4801/2014 lost a site A glycan through N144S (– CHO) while acquiring N158 glycosylation in site B; however, hierarchical site B immunodominance persisted [22,26]. In the past 5 years, multiple glycans on the globular head have been lost at positions 158 (subclade G; 2021–23), 122 (J.2; 2023–25) and 133 (J.2.4; 2024/25), while a putative glycosylation site was recently regained through S144N (+ CHO) substitution among subclade K viruses [27]. Currently, both the 2025/26 J.2 vaccine and circulating subclade K viruses expose site A through shared N122D (– CHO), while differing with respect to site A glycosylation due to T135K (– CHO) and S144N (+ CHO) substitutions in subclade K. Deep mutational scanning of human sera from 2020 suggests a more prominent role for the disrupted N122 glycan, increasing neutralisation sensitivity notably in middle-aged adults, but not teens or children [28]. Notwithstanding antigenic site B mutations, loss of glycosylation within both the J.2 vaccine and subclade K viruses mutually exposes antigenic site A, previously shielded since 1997 and now more accessible to vaccine response.

Because A(H3N2) viruses, including subclade K, have recently accumulated glycosylation changes, we anchored our initial age-related exploration on that basis, recognising more detailed investigations are required end-of-season. The relative under-representation of subclade K cases (vs controls) among participants born pre-1997 suggests pre-existing protection that may in part reflect their greater likelihood of imprinted pre-immunity towards exposed antigenic site A. In 2022/23 we also observed under-representation of the same birth cohorts among A(H3N2) cases, particularly those due to clade 2a.2 bearing similar T135K/A loss of N133 glycosylation, but not other clades retaining the N133 glycan [29]. Conversely, the relative over-representation of subclade K cases among participants born since 1997 may align with their greater antigenic site B orientation, susceptibility to site B mutations, and relative lack of other accumulated and potentially compensatory pre-immunity. Deep mutational scanning reveals prominent neutralisation escape associated with mutations at site B position 189, especially in children and teens, reinforcing their particular vulnerability, but with less clear effects of site B residues 158–160 also mutated in subclade K [28,30]. Because VE is a relative measure of protection in vaccinated compared with unvaccinated individuals, robust pre-existing immunity among unvaccinated participants may also paradoxically dilute or diminish VE estimates, a phenomenon that has been somewhat under-explored for influenza although more recently considered in relation to COVID-19 VE interpretation [29,31-33].

If pre-immunity affects VE estimation, then other more conserved viral targets (e.g. other HA head antigenic sites C to E, HA stalk, neuraminidase) or immunological components (e.g. B-cell or T-cell-mediated), not directly assessed through conventional serological assays, may also require consideration, including alongside prior vaccination and infection history. On an ecological basis, we note that among subtyped viruses in 2024/25, the relative A(H3N2) vs A(H1N1)pdm09 contribution was lower in England (ca 15:85) than in Canada (ca 30:70), elsewhere in Europe (ca 35:65), or the United States (ca 50:50) [34]. The extent to which regional differences in recent influenza circulation and pre-immunity profiles may explain VE variation warrants fuller evaluation.

Following two successive seasons of A(H1N1)pdm09 predominance in 2023/24 (ca 80% of subtyped viruses) and 2024/25 (ca 70% of subtyped viruses) in Canada [11], A(H1N1)pdm09 comprised a minority (< 15%) of our subtyped influenza A viruses in 2025/26. Our 2025/26 mid-season VE estimate against A(H1N1)pdm09 (31%) is lower than any of our prior six mid-season estimates since 2013/14, ranging 44–74%, including 2023/24 (63%) and 2024/25 (53%) [13]. Early 2025/26 A(H1N1)pdm09 VE estimates from Europe are even lower (16%) [15]. Unlike 2023/24 and 2024/25, when clades 5a.2a and 5a.2a.1 co-circulated, virtually all 2025/26 viruses are clade 5a.2a.1. Our capacity to further investigate A(H1N1)pdm09 findings is currently limited by sample size but may also be pursued end-of-season.

Limitations of this study include wide CIs with stratification. We cannot rule out the effects of residual bias and confounding, and cross-study comparisons require consideration of methodological and epidemiological differences including participant profiles (e.g. relative weighting by age), settings (e.g. inpatient or outpatient), analysis periods, vaccines (e.g. inactivated or live attenuated, cell- or egg-based), viruses (e.g. relative subtype and subclade contribution) and clinical outcomes. We sequenced half of all A(H3N2) and A(H1N1)pdm09 case viruses but these may not be representative of the remainder. Hypotheses articulated in this interim analysis focus on A(H3N2) glycosylation effects but other more general subtype-related and also more granular epitope-specific considerations, as well as age-related differences in accumulated influenza exposures, are also likely to apply alone or in combination. We have preliminarily explored age stratification but will undertake more thorough examination of potential imprinting and pre-immunity effects, such as through splining analyses, incorporating multi-season comparisons also as indicated.

## Conclusions

The Canadian SPSN estimates that the 2025/26 influenza vaccine reduced the risk of medically-attended influenza A(H3N2) illness, including due to subclade K, by about 40% overall. Such vaccine protection may be inconsistent with genetic and antigenic indicators of substantial vaccine mismatch, but age-related variation in participant profiles signals a potential role for pre-existing immunity as an effect modifier. Interim findings reinforce the importance of incorporating immuno-epidemiological data into influenza risk assessment, vaccine strain selection, and VE interpretation.

## Data Availability

Sequencing data for included viruses that met provincial/national criteria for upload and their submitting/contributing laboratories can be found on GISAID using the Epi Set ID: EPI_SET_260202sf (https://doi.org/10.55876/gis8.260202sf).

## Supplementary Data

366000 SupplementaryMaterial
