# Supplementary material for: Interim 2025/26 influenza vaccine effectiveness estimates with immuno-epidemiological considerations for A(H3N2) subclade K protection, Canada, January 2026
Source: Euro Surveill. 2026 Feb 5;31(5):2600068. doi: 10.2807/1560-7917.ES.2026.31.5.2600068 (PMC12881843; doi:10.2807/1560-7917.ES.2026.31.5.2600068)
Supplement: SupplementaryMaterial [file 26-00068_Supplementary_material.pdf]

This supplementary material is hosted by *Eurosurveillance* as supporting information alongside the article *Interim 2025/26 influenza vaccine effectiveness estimates with immuno-epidemiological considerations for A(H3N2) subclade K protection, Canada, January 2026*, on behalf of the authors, who remain responsible for the accuracy and appropriateness of the content. The same standards for ethics, copyright, attributions and permissions as for the article apply. Supplements are not edited by *Eurosurveillance* and the journal is not responsible for the maintenance of any links or email addresses provided therein.

Table of Contents

Supplementary Table S1. Whole genome sequencing platforms and quality control metrics used for inclusion of hemagglutinin sequencing data in analysis, by sequencing laboratory....... 3

Supplementary Table S2. Genetic distribution of Influenza A case viruses (n=2092) included in vaccine effective analyses, Canadian Sentinel Practitioner Surveillance Network, 26 October 2025 – 10 January 2026 (epi-weeks 44-01)...... 4

Supplementary Table S3. Participant profile, influenza A(H1N1)pdm09 analyses, Canadian Sentinel Practitioner Surveillance Network, 26 October 2025 – 10 January 2026 (epi-weeks 44–01) (n=3016)....... 5

Supplementary Table S4. Participant profile, influenza A analyses, Canadian Sentinel Practitioner Surveillance Network, 26 October 2025 – 10 January 2026 (epi-weeks 44–01) (n=4844)....... 6

Supplementary Table S5. Summary of available 2025/26 influenza vaccine effectiveness estimates elsewhere....... 7

References 8

Supplementary Table S1. Whole genome sequencing platforms and quality control metrics used for inclusion of hemagglutinin sequencing data in analysis, by sequencing laboratory.

Laboratory	Origin of samples processed	Sequencing platform	Bioinformatics pipeline(s)	Minimum depth requirement	Minimum coverage requirement
Alberta Provincial Laboratory for Public Health	Alberta	Illumina	nf-fluAB (https://github.com/provlab-bioinfo/nf-fluAB)	20X	90%
Public Health Ontario	Ontario	Illumina [1]	CFIA-NCFAD/nf-flu (https://github.com/CFIA-NCFAD/nf-flu)	30X	90%
Institut National de Santé Publique du Québec	Québec	Illumina	FluViewer (https://github.com/KevinKuchinski/FluViewer) & CFIA-NCFAD/nf-flu	20X	95%[a]
National Microbiology Laboratory	British Columbia, Ontario, Québec	Nanopore [2,3]	CFIA-NCFAD/nf-flu	50X	95%

[a] Sequences with coverage between 85-95% meeting the depth requirement are manually investigated for inclusion

Supplementary Table S2. Genetic distribution of Influenza A case viruses (n=2092) included in vaccine effective analyses, Canadian Sentinel Practitioner Surveillance Network, 26 October 2025 – 10 January 2026 (epi-weeks 44-01).

Genetic clade, as defined by ECDC [4] based upon specific HA amino acid substitutions[a]<br>+ additional substitutions by subclade or uniquely identified (antigenic site)	NextStrain subclade [6,7]	BC	Alberta	Ontario	Québec	TOTAL
**Influenza A(H3N2), N (case viruses)**<br>Case viruses successfully sequenced, n (% n/N)		**166**<br>**123 (74%)**	**238**[b]<br>**173 (73%)**	**1032**<br>**414 (40%)**	**260**[c]<br>**125 (48%)**	**1696**[b,c]<br>**835 (49%)**
**2a.3a.1 = 2a.3a + I140K (A) + I223V**	**J**	**16 (13%)**	**12 (7%)**	**64 (15%)**	**14 (11%)**	**106 (13%)**
+ N122D (A)(-CHO) + K276E (C)	J.2				6	6
+ S124N (A)	J.2.2	1		2		3
+ E83D (E) + I214T (D) + HA2: V18M		2	9			11
+ N158K (B) + K189R (B) + HA2: S49N	J.2.3		1			1
+ S54N (C) + G78S (E) + S145N (A) + N216H (D)		2	1		5	8
+ T135K (A)(RBS)(-CHO) + K189R (B)	J.2.4				1	1
+ S144N (A)(+CHO) + N158D (B) + I160K (B)		11	1	62	2	76
**J.2.4 + K2N + S144N (A)(+CHO) + N158D (B) + I160K (B) + Q173R (D)**	**K**	**107 (87%)**	**161 (93%)**	**350 (85%)**	**111 (89%)**	**729 (87%)**
+ N144D (A)(-CHO) + K278E (C)		1	29			30
+ S145N (A)		5		3	1	9
+ S198P (B)				1	7	8
+ V309I (C)		1	4	3		8
**Influenza A(H1N1)pdm09, N (case viruses)**<br>Case viruses successfully sequenced, n (% n/N)		**38**<br>**31 (82%)**	**39**<br>**35 (90%)**	**109**<br>**29 (27%)**	**78**[c]<br>**40 (51%)**	**264**[c]<br>**135 (51%)**
**5a.2a = 5a.2 + K54Q + A186T (Sb) + Q189E (Sb) + E224A (RBS) + E259K + K308R**	**C.1**			**1 (3%)**	**1 (2%)**	**2 (1%)**
+ T120A + K169Q (Ca1) + S83P + HA2: I91V + I183T	C.1.9.3			1	1	2
**5a.2a.1 = 5a.2a + P137S (Ca2) + K142R (Ca2) + D260E + T277A + HA2: E29D + I91V + N124H**	**C.1.1**	**31 (100%)**	**35 (100%)**	**28 (97%)**	**39 (98%)**	**133 (99%)**
+ T216A + T120A + HA2: I45V + I133T + V193A	D.3.1	19	6	6	6	37
+ R113K + A139D (Ca2) + E283K + K302E	D.3.1.1	10	15	16	31	72
+ D139N (Ca2)		2	11	2	2	17
+ R205K (Ca1)			3	4		7

BC, British Columbia; ECDC, European Centre for Disease Prevention and Control; RBS, receptor binding site.

A(H3N2) colour coding aligns with Figure 1 of the main manuscript. Additional substitutions included if located in antigenic or receptor binding sites or involved in gain or loss of glycosylation AND present in ≥1% of A(H3N2) sequenced viruses or ≥2% of A(H1N1)pdm09 sequenced viruses.

[a] Influenza A(H3N2) substitutions are relative to A/Massachusetts/18/2022 (EPI_ISL_16998756) 2a.3a.1 (subclade J) reference virus and A(H1N1)pdm09 substitutions are relative to A/Wisconsin/588/2019 (EPI_ISL_404460) 5a.2 (subclade C) reference virus [5]
[b] Includes one virus with influenza A(H3N2) + influenza B co-infection.
[c] Includes one virus with influenza A(H3N2) + influenza A(H1N1)pdm09 co-infection; counted twice. Specimen sequenced as A(H1N1)pdm09 subclade D.3.1.

Supplementary Table S3. Participant profile, influenza A(H1N1)pdm09 analyses, Canadian Sentinel Practitioner Surveillance Network, 26 October 2025 – 10 January 2026 (epi-weeks 44–01) (n=3016).

Characteristics	All ARI participants (column %, unless otherwise specified)						Influenza vaccinated[a] (row %,)					
	Overall		Influenza A(H1N1)pdm09 cases		Influenza controls		Overall		Influenza A(H1N1)pdm09 cases		Influenza controls	
	n	%	n	%	n	%	n	%	n	%	n	%
N (row %)	3016	100	264	9	2752	91	794	26	55	21	739	27
**Age group (years)** [b]												
1–8	422	14	36	14	386	14	74	18	3	8	71	18
9-17	268	9	35	13	233	8	28	10	2	6	26	11
18–49	1124	37	80	30	1044	38	208	19	14	18	194	19
50–64	560	19	59	22	501	18	147	26	9	15	138	28
≥ 65	642	21	54	20	588	21	337	52	27	50	310	53
Median (IQR)	41 (20-62)		41 (16-62)		41 (20-62)		61 (36-72)		64 (38-72)		60 (36-72)	
**Sex**												
Female	1848	61	142	54	1706	62	516	28	32	23	484	28
Male	1153	38	121	46	1032	38	278	24	23	19	255	25
Unknown	15	0	1	0	14	1	0	0	0	0	0	0
**Comorbidity**[c]												
No	2137	71	198	75	1939	70	423	20	34	17	389	20
Yes	721	24	57	22	664	24	308	43	19	33	289	44
Unknown	158	5	9	3	149	5	63	40	2	22	61	41
**Province**												
Alberta	358	12	39	15	319	12	108	30	8	21	100	31
British Columbia	617	20	38	14	579	21	222	36	13	34	209	36
Ontario	1374	46	109	41	1265	46	381	28	26	24	355	28
Quebec	667	22	78	30	589	21	83	12	8	10	75	13
**Weeks of specimen collection, 2025/26**[d]												
44-45	332	11	16	6	316	11	26	8	2	13	24	8
46-47	456	15	27	10	429	16	78	17	3	11	75	17
48-49	645	21	55	21	590	21	182	28	12	22	170	29
50-51	824	27	101	38	723	26	239	29	17	17	222	31
52-01[e]	759	25	65	25	694	25	269	35	21	32	248	36

ARI, acute respiratory illness; IQR, interquartile range; NA, not applicable.

[a] Vaccination status based on participant or guardian report. Participants vaccinated< 2 weeks before onset of symptoms or with unknown vaccination status or timing were excluded. Only trivalent formulations were used in Canada with virtually all publicly-funded vaccines in SPSN provinces being inactivated (≥99% overall; <5% live-attenuated in BC and Quebec) and egg-based (≥90% overall; <30% cell-based in Alberta and <5% in Ontario). In all provinces, adjuvanted vaccines were available for community-dwelling older adults (≥65 years, ≥75 years in Quebec with high dose vaccines also offered in Ontario.

[b] Children < 1 year excluded based on variability and/or uncertainty in their age-related vaccine eligibility over the course of the epidemic. Older age strata defined as per usual SPSN analyses predicated upon higher likelihood of chronic comorbidity at ≥ 50 years and higher age-associated risk among adults ≥ 65 years [8].

[c] Includes chronic comorbidities that place individuals at higher risk of serious complications from influenza as defined by Canada's National Advisory Committee on Immunization [8].

[d] Missing specimen collection dates were imputed as the date the specimen was received and processed at the laboratory minus 2 days.

[e] Includes epi-weeks 52, 53, and 01.

Supplementary Table S4. Participant profile, influenza A analyses, Canadian Sentinel Practitioner Surveillance Network, 26 October 2025 – 10 January 2026 (epi-weeks 44–01) (n=4844).

Characteristics	All ARI participants (column %, unless otherwise specified)						Influenza vaccinated[a] (row %)					
	Overall		Influenza A cases		Influenza controls		Overall		Influenza A cases		Influenza controls	
	n	%	n	%	n	%	n	%	n	%	n	%
N (row %)	4844	100	2092	43	2752	57	1125	23	386	18	739	27
**Age group (years)** [b]												
1–8	842	17	456	22	386	14	133	16	62	14	71	18
9-17	656	14	423	20	233	8	67	10	41	10	26	11
18–49	1734	36	690	33	1044	38	289	17	95	14	194	19
50–64	746	15	245	12	501	18	182	24	44	18	138	28
≥ 65	866	18	278	13	588	21	454	52	144	52	310	53
Median (IQR)	35 (13-58)		27 (10-49)		41 (20-62)		58 (31-72)		47 (16-71)		60 (36-72)	
**Sex**												
Female	2854	59	1148	55	1706	62	703	25	219	19	484	28
Male	1970	41	938	45	1032	38	421	21	166	18	255	25
Unknown	20	0	6	0	14	1	1	5	1	17	0	0
**Comorbidity**[c]												
No	3581	74	1642	78	1939	70	630	18	241	15	389	20
Yes	1019	21	355	17	664	24	407	40	118	33	289	44
Unknown	244	5	95	5	149	5	88	36	27	28	61	41
**Province**												
Alberta	597	12	278	13	319	12	150	25	50	18	100	31
British Columbia	784	16	205	10	579	21	258	33	49	24	209	36
Ontario	2533	52	1268	61	1265	46	613	24	258	20	355	28
Quebec	930	19	341	16	589	21	104	11	29	9	75	13
**Weeks of specimen collection, 2025/26**[d]												
44-45	347	7	31	1	316	11	26	7	2	6	24	8
46-47	574	12	145	7	429	16	88	15	13	9	75	17
48-49	1063	22	473	23	590	21	261	25	91	19	170	29
50-51	1726	36	1003	48	723	26	404	23	182	18	222	31
52-01[e]	1134	23	440	21	694	25	346	31	98	22	248	36

ARI, acute respiratory illness; IQR, interquartile range; NA, not applicable.

[a] Vaccination status based on participant or guardian report. Participants vaccinated< 2 weeks before onset of symptoms or with unknown vaccination status or timing were excluded. Only trivalent formulations were used in Canada with virtually all publicly-funded vaccines in SPSN provinces being inactivated (≥99% overall; <5% live-attenuated in BC and Quebec) and egg-based (≥90% overall; <30% cell-based in Alberta and <5% in Ontario). In all provinces, adjuvanted vaccines were available for community-dwelling older adults (≥65 years, ≥75 years in Quebec with high dose vaccines also offered in Ontario.

[b] Children < 1 year excluded based on variability and/or uncertainty in their age-related vaccine eligibility over the course of the epidemic. Older age strata defined as per usual SPSN analyses predicated upon higher likelihood of chronic comorbidity at ≥ 50 years and higher age-associated risk among adults ≥ 65 years [8]

[c] Includes chronic comorbidities that place individuals at higher risk of serious complications from influenza as defined by Canada's National Advisory Committee on Immunization [8].

[d] Missing specimen collection dates were imputed as the date the specimen was received and processed at the laboratory minus 2 days.

[e] Includes epi-weeks 52, 53, and 01.

Supplementary Table S5. Summary of available 2025/26 influenza vaccine effectiveness estimates elsewhere.

	**England** (epi-weeks 40-44) [9]	**England** (epi-weeks 40-44) [9]	**Europe**[a] (epi-weeks 41-49) [10]	**Canada** (epi-weeks 44-01[b])
**Outcome**	Any ED visit	Any hospitalization	ARI-related outpatient visit	ARI-related outpatient visit
**Sample size, influenza A** (n vaccinated / N [%])	331 / 4772 (7%) cases<br>2850 / 24017 (12%) controls	257 / 3396 (8%) cases<br>2467 / 20658 (12%) controls	866 cases<br>4165 controls	386 / 2092 (18%) cases<br>739 / 2752 (27%) controls
**Proportion A(H3N2)** (% subtyped)	85%<br>(19% subtyped)	85%<br>(25% subtyped)	75%<br>(85% subtyped)	86%<br>(94% subtyped)
**Proportion subclade K** (% sequenced)	87% between weeks 35-43<br>(Not reported)		Not reported	87%<br>(49% sequenced)
**Adjusted for**	Week, age, region, clinical risk status		Onset date, age, sex, chronic condition	Week, age, province [c]
**Influenza A VE**	-	-	44% (25, 59) [d]	38% (27, 47)
**≤ 17 years**	75% (52, 88)	74% (63, 82)	-	35% (10, 54)
**18-64 years**	33% (13, 49)	33% (10, 50)	-	46% (32, 57)
**≥ 65 years**	35% (22, 45)	39% (26-50)	-	21% (−8, 42)
**Influenza A(H3N2) VE**	-	-	52% (29, 69)	40% (28, 49)
**≤ 17 years**	75% (52, 88)	73% (48, 87)	52% (21, 72)	36% (10, 55)
**18-64 years**	60% (15, 85)	66% (24, 88)	57% (4, 84)	48% (33, 60)
**≥ 65 years**	35% (-9, 63)	32% (-14, 61)	-	25% (−7, 48)
**Influenza A(H1N1)pdm09 VE**	-	-	16% (-43, 54)	31% (3, 50)

ARI, acute respiratory illness; ED, emergency department; epi-weeks, epidemiological weeks; VE, vaccine effectiveness.

Additional 2025/26 interim VE estimates not shown above from Beijing (owing to author expression of concern pending resolution) [11], nor from France or Scotland owing to lack of subtype-specific VE estimates [12,13].

[a] Includes France, Germany, Ireland, Italy, the Netherlands, Portugal, Spain, Romania
[b] Includes epi-week 53
[c] Adjustment for sex and/or comorbidity had minimal impact (≤ 5% absolute) on VE findings, greater for influenza A VE in ≥ 65 year olds (i.e., further reduced by 8%)
[d] VE against any influenza (includes 1 case of influenza B, 865 cases of influenza A)

References

1. Public Health Ontario. Influenza Genomic Surveillance in Ontario: 2025-26 Early Season [Internet]. 2025 Dec [cited 2026 Jan 15]. Available from: https://www.publichealthontario.ca/-/media/Documents/I/24/influenza-genomic-surveillance-ontario.pdf?rev=61de388cb2ed42c89a61732f0c19f57f&sc_lang=en&hash=F4B955708090505183DC0DE8ED53E26B

2. Zhou B, Donnelly ME, Scholes DT, St. George K, Hatta M, Kawaoka Y, et al. Single-Reaction Genomic Amplification Accelerates Sequencing and Vaccine Production for Classical and Swine Origin Human Influenza A Viruses. Journal of Virology. 2009 Oct;83(19):10309–13.

3. Gao R, Irvine K, Buchanan C, Slater C, Toledo NP, Jia J, et al. Optimized respiratory virus influenza A whole genome sequencing strategies for improving even read coverage of segments. Journal of Clinical Virology. 2026 Feb 1;182:105904.

4. European Centre for Disease Prevention and Control. Influenza virus characterization - Summary Europe, May 2024 [Internet]. Stockholm: ECDC; 2024 May. Available from: https://www.ecdc.europa.eu/en/publications-data/influenza-virus-characterization-summary-europe-may-2024

5. Shu Y, McCauley J. GISAID: Global initiative on sharing all influenza data – from vision to reality. Eurosurveillance [Internet]. 2017 Mar 30 [cited 2025 Jan 27];22(13). Available from: https://www.eurosurveillance.org/content/10.2807/1560-7917.ES.2017.22.13.30494

6. Neher RA, Huddleston J, Bedford T, Lewis NS, Harvey R, Galiano M, et al. Nomenclature for tracking of genetic variation of seasonal influenza viruses [Internet]. medRxiv; 2025 [cited 2026 Jan 19]. p. 2025.12.06.25341755. Available from: https://www.medrxiv.org/content/10.64898/2025.12.06.25341755v1

7. Aksamentov I, Roemer C, Hodcroft E, Neher R. Nextclade: clade assignment, mutation calling and quality control for viral genomes. JOSS. 2021 Nov 30;6(67):3773.

8. National Advisory Committee on Immunization (NACI). Statement on seasonal influenza vaccines for 2025-26 [Internet]. 2025. Available from: https://www.canada.ca/content/dam/phac-aspc/documents/services/publications/vaccines-immunization/national-advisory-committee-immunization-statement-seasonal-influenza-vaccines-2025-2026/national-advisory-committee-immunization-statement-seasonal-influenza-vaccines-2025-2026.pdf

9. Kirsebom FC, Thompson C, Talts T, Kele B, Whitaker HJ, Andrews N, et al. Early influenza virus characterisation and vaccine effectiveness in England in autumn 2025, a period dominated by influenza A(H3N2) subclade K. Eurosurveillance. 2025;

10. European Centre for Disease Prevention and Control. Early estimates of seasonal influenza vaccine effectiveness against influenza requiring medical attention at primary care level in Europe, week 41 - 49, 2025 [Internet]. 2025 Dec. Available from: https://www.ecdc.europa.eu/en/news-events/early-estimates-seasonal-influenza-vaccine-effectiveness-against-influenza-requiring

11. Shen Y, Zhang D, Feng Z, Ma C, Shi W, Duan W, et al. Moderate protection from vaccination against influenza A(H3N2) subclade K in Beijing, China, September to December 2025. Eurosurveillance. 2026 Jan 15;

12. Clercq AD, Blanquart F, Vieillefond V, Visseaux B, Jacques A, Haim-Boukobza S, et al. Interim vaccine effectiveness against influenza virus among outpatients, France, October 2025 to January 2026. Eurosurveillance. 2026 Jan 15;

13. Public Health Scotland. Viral Respiratory Diseases in Scotland Surveillance Report [Internet]. 2025 Dec. Available from: https://publichealthscotland.scot/media/36525/week-49-11-12-25-viral-respiratory-diseases-in-scotland-surveillance-report.pdf
